# Is chemically dispersed oil more toxic to Atlantic cod (*Gadus morhua*) larvae than mechanically dispersed oil? A transcriptional evaluation

**DOI:** 10.1186/1471-2164-13-702

**Published:** 2012-12-14

**Authors:** Pål A Olsvik, Kai K Lie, Trond Nordtug, Bjørn Henrik Hansen

**Affiliations:** 1 National Institute of Nutrition and Seafood Research, Nordnesboder 1-2, Bergen, N-5005, Norway; 2 SINTEF, Materials and Chemistry, Marine Environmental Technology, Trondheim, N-7465, Norway

**Keywords:** Atlantic cod larvae, Exposure, Chemical, Natural oil dispersion, Transcription

## Abstract

**Background:**

The use of dispersants can be an effective way to deal with acute oil spills to limit environmental damage, however very little is known about whether chemically dispersed oil have the same toxic effect on marine organisms as mechanically dispersed oil. We exposed Atlantic cod larvae to chemically and mechanically dispersed oil for four days during the first-feeding stage of development, and collected larvae at 14 days post hatch for transcriptional analysis. A genome-wide microarray was used to screen for effects and to assess whether molecular responses to chemically and mechanically dispersed oil were similar, given the same exposure to oil (droplet distribution and concentration) with and without the addition of a chemical dispersant (Dasic NS).

**Results:**

Mechanically dispersed oil induced expression changes in almost three times as many transcripts compared to chemically dispersed oil (fold change >+/−1.5). Functional analyses suggest that chemically dispersed oil affects partly different pathways than mechanically dispersed oil. By comparing the alteration in gene transcription in cod larvae exposed to the highest concentrations of either chemically or mechanically dispersed oil directly, the chemically dispersed oil affected transcription of genes involved nucleosome regulation, i.e. genes encoding proteins participating in DNA replication and chromatin formation and regulation of cell proliferation, whereas the mechanically dispersed oil most strongly affected genes encoding proteins involved in proteasome-mediated protein degradation. *Cyp1a* was the transcript that was most strongly affected in both exposure groups, with a 60-fold induction in the two high-exposure groups according to the RT-qPCR data, but no significant difference in transcriptional levels was observed between the two treatments.

**Conclusions:**

In summary, dispersants do not appear to add to the magnitude of transcriptional responses of oil compounds but rather appear to lower or modify the transcriptional effect on cod larvae.

## Background

Crude oil is a complex mixture of a range of different components like aliphatic and aromatic hydrocarbons (PAHs), phenols, and a substantial amount of unknown compounds [1]. Following an acute oil spill, waves, wind and sunlight will cause weathering of the oil, altering the appearance and composition of the oil dramatically and dynamically [2]. The weathering process generates oil-in-water dispersions, consisting of oil droplets in the water phase. Micron-sized oil droplets will to a minimal degree resurface, and they will be a source of oil compounds for marine organisms through leakage and dissolution, as the chemical equilibrium for oil compounds between the water phase and the oil droplets continuously will vary. Use of dispersants after an acute oil spill, as demonstrated by the extensive use of the dispersant Corexit 9500 during the Deepwater Horizon accident in the Gulf of Mexico in 2010 [3], will increase the amounts of oil droplets in the seawater column. The main purpose of using dispersant is to move the oil into the water column as oil dispersions which will dilute and biodegrade the oil more rapidly than if the oil was left on the surface [4,5]. More knowledge is needed on the fate and effects of oil droplets in the water column in terms of lifetime, adhesion to particles, dissolution rates and not at least their toxicity to sensitive marine organisms.

Fish embryo and larvae are generally regarded to be particularly vulnerable to the toxic compounds in crude oil [6-13]. Exposure studies carried out with pink salmon (*Oncorhynchus gorbuscha*) and pacific herring (*Clupea pallasi*) embryos after the Exxon Valdez accident showed that dissolved PAHs alone (i.e. without oil droplets) are sufficient for toxic impacts [6,8,9]. Studying zebrafish (*Danio rerio*), Carls et al. [8] exposed fish embryos in a physical barrier separating dissolved PAH from oil droplets, and showed comparable biological responses to water containing either dissolved PAH alone, or dissolved PAH plus droplets. We recently evaluated the potential contribution of oil droplets to the toxicity of dispersed oil to first feeding Atlantic cod (*Gadus morhua*) larvae [14], and observed that it was mainly the water-soluble fraction of oil and not the oil droplets themselves that induced altered gene transcription of detoxifying enzymes in the fish larvae. From these studies it appears that oil droplets characteristics do not attribute to the toxic effects of PAH and other components in crude oil to fish embryo and larvae, however, very little is known about whether or not chemically dispersed oil droplets have the same toxic effects as mechanistically dispersed oil droplets on fish larvae. Most literature studies have compared the toxic effects of chemically and mechanically dispersed oil by comparing the toxicity of low energy water accommodated fractions (LE-WAF) or high energy water accommodated fractions (HE-WAF), with chemically-enhanced water accommodated fractions (CE-WAF). Such comparisons may be useful in terms of comparatively testing the impact of the dispersant application, however, in terms of oil exposure, the two approaches differ significantly. Generation of CE-WAF will increase the oil concentration in the water phase and cause formation of very small oil droplets which will persist for a longer period of time in the water phase compared to WSFs generated using the LE-WAF or HE-WAF approach. Often there is no information given on the oil droplet characteristics (e.g. size range, composition, etc.). In other words, dispersions generated with equal energy input with and without dispersant therefore creates dispersions (water-accommodated fractions) differing in oil droplet size distribution, oil concentration and chemical composition of the water soluble fraction. Using chemical dispersant it is the impossible to separate the toxic effects related to the presence of dispersant from secondary effects related to changes in oil concentration and chemical composition. Using static or semi-static systems is expected to further enhance the differences due to size dependent oil droplet surfacing velocity in the two dispersions. One way of overcoming these problems in order to isolate the effect of the oil/dispersant interaction is to compare dispersions with similar oil concentrations and oil droplet size distributions with and without dispersant in a continuous flow system, and this is what has been done in the present work.

The aim of this work was to evaluate whether chemically dispersed (CD) oil, generated so that it was comparable in terms of oil droplet characteristics and concentrations, induce the same transcriptional responses in fish larvae as mechanically dispersed (MD) oil, or whether hydrocarbons in chemically dispersed oil droplets are more toxic due to the way the droplets are formed. Transcriptional responses as a measure of toxicity were studied in Atlantic cod larvae exposed to either chemically or mechanically dispersed oil droplets over a period of four days at the age of 10–14 days post hatch during the first-feeding life stage. The Atlantic cod was selected because it inhabits waters with extensive oil and gas exploration on both sides of the North Atlantic, and also because acute oil spills near spawning grounds may endanger local populations. For transcriptome-wide screening, a Nimblegen microarray containing 135 000 oligos was used. Gene Set Enrichment Analysis (GSEA) and Ingenuity Pathway Analysis (IPA) were applied for functional and pathway analysis. Our hypothesis was that oil droplets should be expected to be equally toxic independent on the way they are generated, and that the use of dispersants does not work additive to the transcriptomic responses.

## Results

### Chemical analysis

**Figure 1:**
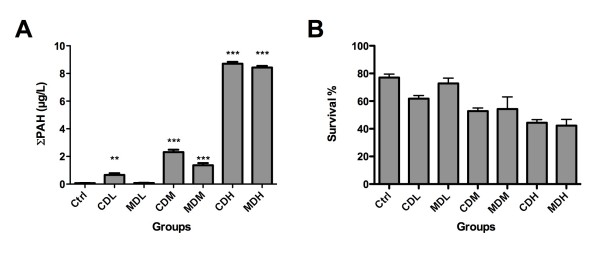
**PAH exposure concentrations and survival.A**) ∑PAH concentrations in the different exposure media (average ± SEM, n = 8). **B**) Survival (%) after 4 days exposure (at day 14) in the different treatments (mean ± SEM, n = 4 throughout).

**Figure 2:**
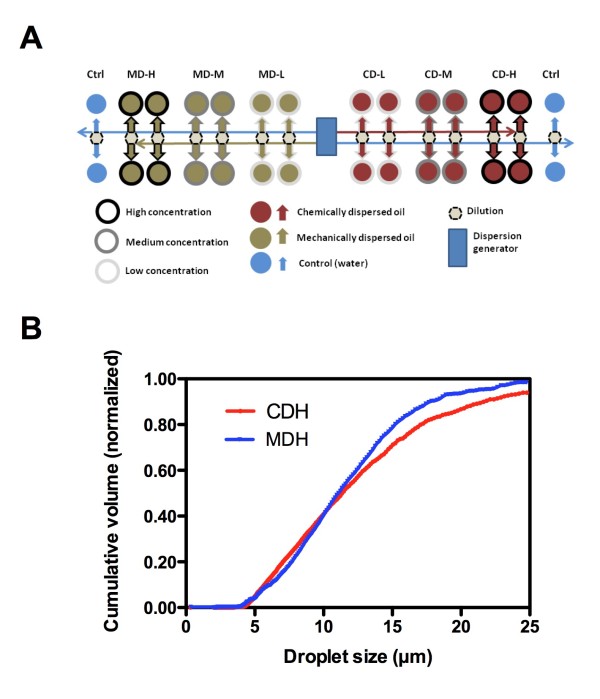
**A) Schematic diagram of the experimental design.** For both mechanically dispersed (MD) and chemically dispersed (CD) oil, 3 concentrations – low (L), medium (M) and high (H) – were used (n = 4 for all). Dispersions were made using two dispersant generators where oil-in-water-dispersions were generated from either oil premixed with dispersant, or from oil alone. Dilution of the dispersions was computer controlled using solenoid vales to collect defined ratios of dispersions and clean seawater from the two feed tubes running along the exposure chambers. A total of 4 control chambers were used. **B**) Cumulative size distribution based on volume of oil droplets recorded at the outlet of the high exposure vessels with mechanically and chemically dispersed oil. Data in each group are average of 4 parallel vessels. Algae with mean size of 4.7 μm have been subtracted.

The experimental setup is shown in Figure 1A, while Figure 1B shows the averaged values of cumulative size distributions (volume based) recorded at the outlet of the exposure vessels from the high exposure groups. Figure 2A shows the exposure concentrations of ∑PAHs. Separations between naphthalenes, 2–3 ring PAHs and 4–6 ring PAHs are presented for all exposure groups (Additional file 1). These concentrations are average of 8 samples analyzed by GC/MS (mean±SEM). Except for the MDL group, the ∑PAH were significantly higher in the exposure groups compared to the control (one-way ANOVA, Dunnett’s posthoct test). The analyses also showed that the ∑PAH levels were significantly higher in the low and medium concentration groups of chemically dispersed oil than in mechanically dispersed oil (one-way ANOVA, Newman Keuls posthoc test, P<0.001). Thus, relatively comparable treatments of cod larvae with chemically and mechanically dispersed oil with respect to oil concentrations were obtained. Similar size distributions of oil droplets for both dispersion types were confirmed with the particle characterization.

### Larvae survival

In general the mortality during the first feed period of cod larvae is expected to be high and the highest survival rate was 77.0% (± 5.4) in the controls. The average lethality in all exposed groups was higher than that of the control groups and the lethality increased with increasing exposure concentration (Figure 2B).

### Microarray screening

**Figure 3:**
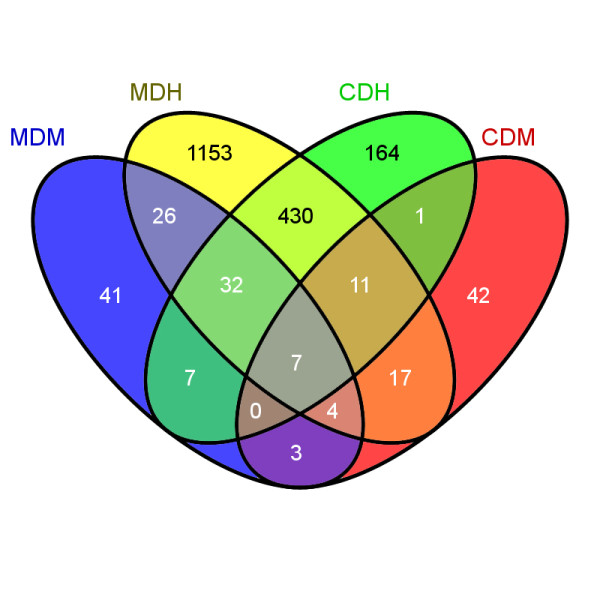
**Venn diagram.** Number of transcripts significantly differentially expressed in Atlantic cod larvae exposed to chemically or mechanically dispersed oil for 4 days and sampled at 14 days post hatch. MDM = mechanically dispersed oil - medium concentration. MDH = mechanically dispersed oil - high concentration. CDM = chemically dispersed oil - medium concentration. CDH = chemically dispersed oil - high concentration.

ANOVA analysis of the microarray data yielded gene lists with 16, 85 and 652 significant affected genes in the low (CDL), medium (CDM) and high (CDH) groups of Atlantic cod larvae exposed to chemically dispersed oil, respectively (P<0.05). The affected genes in cod larvae exposed to mechanically dispersed oil contrasted against the control were 33 (MDL), 120 (MDM) and 1680 (MDH) genes, respectively. Figure 3 shows a Venn diagram of the number of overlapping genes between the different exposure groups. Based solely on the numbers of affected genes in the high exposure groups, the result indicates that oil dispersion that were mechanically dispersed mediated greater changes in gene transcription to the larvae than chemically dispersed oil. Surprisingly few common genes were observed between the two high-exposure groups; only 480 common genes were observed in the MDH and CDH groups. The four groups exposed to the highest concentrations (CDM, CDH, MDM and MDH) shared only seven common genes, and all of these with annotations were related to the cytochrome P450 system (*cyp1a*, *cyp1b1* (2X), *cyp1c1*, *ahrr* and two oligo sequences with unknown identity).

Additional file 2 shows the gene lists generated with the ANOVA analysis from the six groups of larvae (CDH, CDM, CDL, MDH, MDM and MDL), with sequence IDs, sequence descriptions, gene names used for functional analysis, P-values and fold changes. *Cyp1a* showed the strongest response in the larvae exposed to the highest concentrations of dispersed oil. According to the microarray data, *cyp1a1* was 12.6-fold up-regulated in larvae from the CDH group, whereas *cyp1b1* was 10.3-fold up-regulated. *cyp1a1* and *cyp1b1* were 17.6-fold and 16.8-fold up-regulated, respectively, in larvae from the MDH group. *cyp1a1* and *cyp1b1* were also significantly up-regulated in cod larvae from the two medium concentration exposure groups, CDM and MDM. In larvae from the first group, *cyp1a1* was 8.4-fold up-regulated, while *cyp1b1* was 4.7-fold up-regulated. In larvae from the MDM group *cyp1a1* was 10.1-fold up-regulated, while *cyp1b1* showed a 6.0-fold up-regulation. A still significant up-regulation of *cyp1a1* (2.7-fold) was observed in cod larvae exposed to the lowest concentration of chemically dispersed oil droplets (CDL), but not in larvae exposed to the lowest concentration of mechanically dispersed oil droplets (MDL). In other words, based on the number of significantly differentially expressed transcripts and induction of the well-established biomarker *cyp1a*, the microarray data suggest that mechanically dispersed oil was slightly more toxic to the fish larvae compared to the chemically dispersed oil. Also the data for the third most differentially regulated transcript in larvae from the CDH and MDH exposure groups, the aryl hydrocarbon receptor repressor (*ahrr*), points in the same direction. *Ahrr* were 7.0-fold and 4.7-fold up-regulated in larvae from the MDH group (two different probes), according to two oligo sequences both annotated to this gene, whereas *ahrr* was significantly but only 2.8-fold up-regulated in larvae from the corresponding CDH group.

Less coherent results were obtained for the transcripts showing the highest degree of down-regulation. In the cod larvae exposed to the highest concentration of chemically dispersed oil (CDH), centromere protein i (−3.9-fold), DEAH (asp-glu-ala-his) box polypeptide 35 (−3.8-fold), and timeless interacting protein (−3.7-fold) showed the strongest down-regulation (Additional file 2). In cod larvae exposed to the highest concentration of mechanically dispersed oil (MDH), cell division cycle associated 7 (−13.4-fold), hemopexin (*hpx*, or warm temperature acclimation-related 65 kDa protein (*wap65*) (−11.4-fold), and chromosome 6 open reading frame 58 (−8.7-fold) showed the strongest down-regulation response (Additional file 2). Again, based on the degree of transcription fold changes, the microarray data suggest that mechanically dispersed oil mediated a slightly stronger response than chemically dispersed oil.

### RT-qPCR analysis

**Figure 4:**
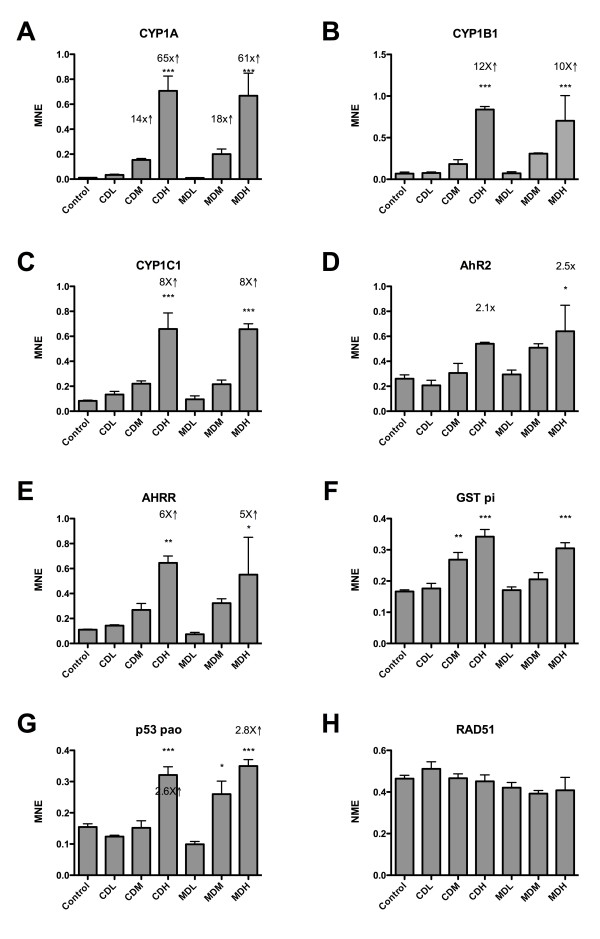
**Transcriptional levels of a selected number of genes analyzed with RT**-**qPCR.A**) CYP1A, **B**) CYP1B1, **C**) CYP1C1, **D**) AHR2, **E**) AHRR, **F**) GST π, **G**) p53, and **H**) RAD51. All groups n = 4 except MDM and MDH n = 3. MNE = mean normalized expression. The raw Ct values were transformed to quantities and PCR efficiency corrected according to the *geNorm* manual, and divided by the normalization factor calculated from four reference genes by *geNorm*. Arrows and numerals show fold changes. * P<0.05, **P<0.01. ***P<0.001.

**Figure 5:**
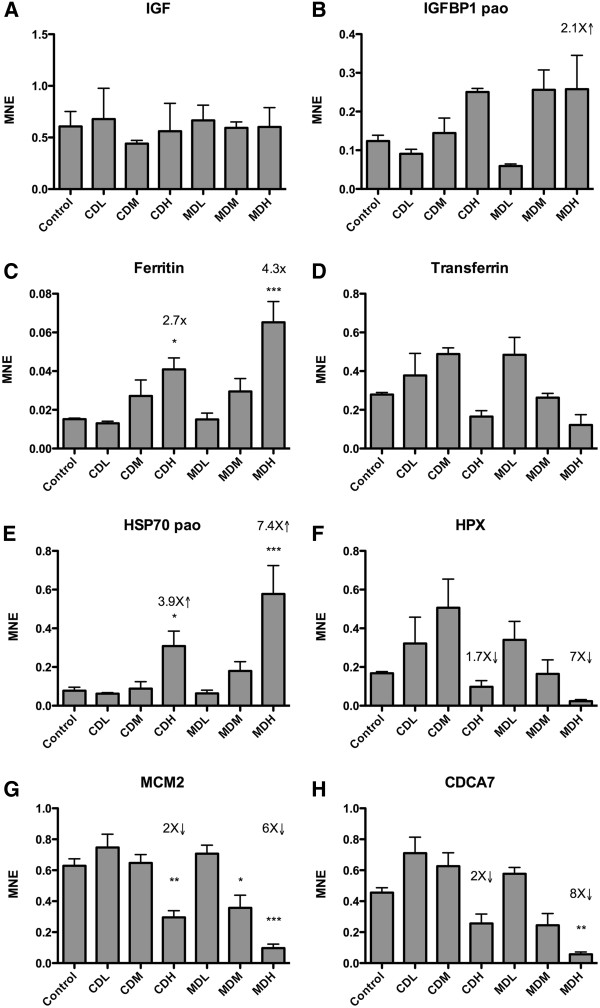
**Transcriptional levels of a selected number of genes analyzed with RT**-**qPCR.A**) IGF, **B**) IGFBP1, **C**) ferritin, **D**) transferrin, **E**) HSP70, **F**) HPX (WAP65-2), **G**) MCM2, and **H**) CDCA7. All groups n = 4 except MDM and MDH n = 3. MNE = mean normalized expression. The raw Ct values were transformed to quantities and PCR efficiency corrected according to the *geNorm* manual, and divided by the normalization factor calculated from four reference genes by *geNorm*. Arrows and numerals show fold changes. * P<0.05, **P<0.01. ***P<0.001.

In order to verify the microarray results, a set of transcripts were evaluated by RT-qPCR. In general, the quantitative real-time qPCR results were in line with the microarray data. Based on 9 quantified transcripts showing significant effect analyzed with RT-qPCR, the correlation between the microarray data and RT-qPCR was r=0.99 for the CDH group and r=0.98 for the MDH group (Spearman rank correlation, P<0.0001). Figures 4 and 5 show the transcriptional levels of 16 genes quantified with RT-qPCR. Of the evaluated transcripts, *cyp1a* showed to strongest response with a 64.9-fold induction in larvae from the CDH group and a 61.3-fold induction in larvae from the MDH group (Figure 4). In the medium-exposure groups, *cyp1a* showed a 14.1-fold induction in larvae from the CDM group, and 18.4-fold induction in larvae from the MDM group. RT-qPCR data for a set of evaluated transcripts and their significance are shown in Figure 2. Also *cyp1b1* (Figure 4B) and *cyp1c1* (Figure 4C) showed significant responses to dispersed oil exposure, with *cyp1b1* showing a stronger response than *cyp1c1* in the two high-exposure groups. The *ahrr* transcript (Figure 4E) was more strongly affected than the *ahr2* transcript (Figure 4D). The significant up-regulation of *gst π* (Figure 4F) suggests that phase II metabolism was affected in the cod larvae, while altered transcription of *p53* (Figure 4G) suggest that dispersed oil exposure may have mediated an effect on DNA integrity. No significant effects of oil exposure were observed on the growth marker *igf* (Figure 5A) or *igfbp1* (Figure 5B). *Ferritin* (Figure 5C) and *hsp70* (Figure 5E) transcription was significantly up regulated by dispersed oil treatment, while *mcm2* (Figure 5G) and *cdca7* (Figure 5H) were significantly down-regulated by the treatment.

### Functional pathway analysis

Gene set enrichment analysis (GSEA) and Ingenuity Pathway Analysis (IPA) was used for functional analyses of the transcriptional data. Additional file 3 shows the top ranked gene sets in larvae from all exposure groups compared to the control group. Table 1 shows the GSEA gene sets significantly affected comparing the two high-exposure groups directly (CDH versus MDH). Only the top-ranked gene sets are shown for each comparison. GSEA of the microarray data showed that most significantly affected gene sets were observed in the cod larvae exposed mechanically dispersed oil droplets (MDH). The overall pattern of affected gene sets in cod larvae in the different exposure groups suggest that the way oil droplets are generated have an effect on toxicity in fish larvae. By comparing the alteration in gene transcription in cod larvae exposed to the highest concentrations of either chemically or mechanically dispersed oil directly, the chemically dispersed oil affected transcription of genes involved nucleosome regulation, i.e. genes encoding proteins participating in DNA replication and chromatin formation and regulation of cell proliferation, whereas the mechanically dispersed oil mainly affected genes encoding proteins involved in proteasome-mediated protein degradation (Additional file 3). Compared to larvae in the control group, the GSEA data showed that mechanically dispersed oil also mediated a general down-regulation of many transcripts in cod larvae in the MDH group.

**Table 1:** **Gene set enrichment analysis** (**GSEA**)

**Group**	**Gene set**	**Size**	**ES**	**NES**	**p value**	**FDR**
**Enriched in CDH**						
1	nucleosome	14	0.76	2.34	0.00	0.84
2	structural constituent of eye lens	12	0.72	2.16	0.00	6.16
3	inner ear morphogenesis	21	0.61	2.15	0.00	4.38
4	nucleosome assembly	21	0.59	2.03	0.00	11.71
5	chromatin assembly	21	0.59	2.03	0.00	9.36
6	intramolecular oxidoreductase activity	16	0.61	1.96	0.01	14.74
7	DNA-dependent DNA replication	19	0.53	1.85	0.00	31.63
8	DNA packaging	24	0.50	1.82	0.00	33.31
9	methylation	43	0.41	1.78	0.00	40.09
10	methyltransferase activity	43	0.41	1.78	0.00	36.08
**Enriched in MDH**						
1	proteasomal ubiquitin-dependent protein catabolic process	60	−0.51	−2.01	0.00	12.63
2	proteasomal protein catabolic process	60	−0.51	−2.01	0.00	6.32
3	ubiquitin-protein ligase activity	71	−0.49	−1.93	0.00	14.41
4	protein ubiquitination	94	−0.46	−1.92	0.00	13.14
5	regulation of protein catabolic process	10	−0.75	−1.92	0.00	10.60
6	protein modification by small protein conjugation	101	−0.45	−1.90	0.00	11.47
7	ubiquitin thiolesterase activity	33	−0.54	−1.88	0.00	13.19
8	thiolester hydrolase activity	42	−0.51	−1.87	0.00	12.49
9	protein modification by small protein conjugation or removal	123	−0.43	−1.87	0.00	11.58
10	membrane raft	65	−0.47	−1.86	0.00	11.47
11	cellular protein catabolic process	129	−0.42	−1.86	0.00	10.68
12	proteolysis involved in cellular protein catabolic process	129	−0.42	−1.86	0.00	9.79

Direct comparison of genes uniquely expressed in cod larvae in the CDH and MDH exposure groups. Size: number of genes in each gene set. ES = enrichment score. NES = normalized enrichment score. Nominal *p* value. FDR = false discovery rate.

IPA was used to evaluate whether or not chemically dispersed oil mediated a different toxic response compared to mechanically dispersed oil. Since IPA only can map mammalian homolog identifiers, GeneCards IDs were submitted for biological function and pathway analysis, using top BlastX hits and assuming orthologous genes have the same function. For example, because fish often have two isoforms of many genes due to genome duplication, labeled A and B, mammalian homolog identifiers had to be used as input for the IPA analysis, without knowing the exact function of the separate teleostean isoforms. The number of mapped IDs for IPA analysis in the different exposure groups were; CDH) 583 out of 652, CDM) 75 out of 85, CDL) 13 out of 16, MDH) 1501 out of 1680, MDM) 101 out of 120 and MDL) 30 out of 33.

According to the IPA Core Analyses (Additional file 4), using a maximum number of 70 molecules in each pathway, the top affected networks in the cod larvae exposed to the highest concentration of chemically dispersed oil (CDH group) were “RNA post-transcriptional modification, cellular assembly and organization, cell morphology” with a score of 98, “DNA replication, recombination, and repair, cell cycle, cancer” with a score of 84 and “Lipid metabolism, molecular transport, small molecule biochemistry” with a score of 65. The corresponding top affected pathways in the cod larvae exposed to mechanically dispersed oil were “RNA post-transcriptional modification, cellular assembly and organization, cell morphology” with a score of 75, “Cellular function and maintenance, small molecule biochemistry, DNA replication, recombination, and repair” with a score of 66, and “Lipid metabolism, small molecule biochemistry, vitamin and mineral metabolism” with a score of 62.

IPA-Tox is a data analysis capability within IPA that delivers a focused toxicity assessment of input molecules using toxicogenomics approaches. Table 2 shows the significant IPA-Tox pathways in the six groups of cod larvae exposed to oil dispersants. The most significantly affected IPA-Tox pathways in the high-exposure chemically dispersed oil data set (CDH) were “Positive Acute Phase Response Proteins”, “Aryl Hydrocarbon Receptor Signaling”, “Cell Cycle: G1/S Checkpoint Regulation”, “Negative Acute Phase Response Proteins”, and “Fatty Acid Metabolism” (Table 2). In the larvae exposed to the highest concentration of mechanically dispersed oil (MDH), the top IPA-Tox list included “Negative Acute Phase Response Proteins”, “p53 Signaling”, “Liver Proliferation”, “Oxidative Stress”, and “Cholesterol Biosynthesis” (Table 2). Fisher’s exact test was used to calculate a p-value determining the probability that the association between the genes in the dataset and the IPA-Tox pathways was explained by chance alone.

**Table 2:** **Mechanistic responses in Atlantic cod larvae exposed to chemically** (**CD**) **or mechanically** (**MD**) **dispersed oil droplets for four days**

**Group**	**Name**	**p**-**value**	**Ratio**	**Group**	**Name**	**p**-**value**	**Ratio**
CDH	Positive Acute Phase Response Proteins	5.1E-05	6/30 (0.2)	MDH	Negative Acute Phase Response Proteins	2.07E-05	5/8 (0.625)
CDH	Aryl Hydrocarbon Receptor Signaling	2.66E-04	12/157 (0.076)	MDH	p53 Signaling	4.86E-04	14/95 (0.147)
CDH	Cell Cycle: G1/S Checkpoint Regulation	3.04E-04	7/57 (0.123)	MDH	Liver Proliferation	7.01E-04	17/133 (0.128)
CDH	Negative Acute Phase Response Proteins	6.08E-04	3/8 (0.375)	MDH	Oxidative Stress	7.68E-04	10/57 (0.175)
CDH	Fatty Acid Metabolism	2.06E-03	9/123 (0.073)	MDH	Cholesterol Biosynthesis	1.13E-03	5/16 (0.312)
CDM	FXR/RXR Activation	3.6E-07	6/87 (0.069)	MDM	Aryl Hydrocarbon Receptor Signaling 4	6.02E-04	5/157 (0.032)
CDM	LXR/RXR Activation	2.28E-06	6/119 (0.05)	MDM	Xenobiotic Metabolism Signaling	7.95E-04	7/349 (0.02)
CDM	Positive Acute Phase Response Proteins	2.52E-06	4/30 (0.133)	MDM	Cytochrome P450 Panel - Substrate is a Xenobiotic (Human)	2.71E-03	2/18 (0.111)
CDM	Fatty Acid Metabolism	6.67E-04	4/123 (0.033)	MDM	NRF2-mediated Oxidative Stress Response	3.69E-03	5/237 (0.021)
CDM	Xenobiotic Metabolism Signaling	8.92E-04	6/349 (0.017)				
				MDL	Negative Acute Phase Response Proteins	3.6E-05	2/8 (0.25)
				MDL	Decreases Respiration of Mitochondria	7.05E-05	2/11 (0.182)
				MDL	LXR/RXR Activation	3.42E-04	3/119 (0.025)
				MDL	TR/RXR Activation	4.45E-03	2/86 (0.023)

IPA Tox lists with significant responses H = high concentration M = medium concentration L = low concentration.

In an attempt to identify unique and common molecules across the gene lists the IPA-Compare function was applied. Additional file 5 shows the associated functions of the top networks as suggested by IPA Core Analysis in significantly affected transcripts in cod larvae exposed to the different exposure treatments. According to the IPA-Tox, the unique molecules in both the CDH and MDH lists encode proteins responding to oxidative stress. “NRF2-mediated Oxidative Stress Response” (2.51E-03) topped the list in larvae from the CDH exposure group, while “Oxidative Stress” (3.31E-05) and “NRF2-mediated Oxidative Stress Response” (2.52E-03) topped the list in the larvae from the MDH group. These results do not suggest that the two different ways of inducing oil droplets has influenced a major difference in affected pathways in the highest exposure concentration groups. In the medium concentration groups, molecules unique to larvae exposed to chemically induced oil (CDM), “LXR/RXR Activation” (8.76E-07) topped the list, followed by “Positive Acute Phase Response Proteins” (1.32E-06) and “FXR/RXR Activation” (9.54E-05), while “PPARa/RXRa Activation” (5.38E-03) topped the MDM group.

Molecules common to the two high-exposure groups, suggests that either way of inducing dispersed oil affected many of the same pathways as indicated by the IPA-Tox lists for the separate exposure groups shown in Table 1. The five most significant pathways according to the common CDH and MDH molecule list were “Negative Acute Phase Response Proteins” (2.59E-04), “Aryl Hydrocarbon Receptor Signaling” (3.75E-04), “Cell Cycle: G1/S Checkpoint Regulation” (4.12E-04), “Positive Acute Phase Response Proteins” (1.61E-03) and “Cholesterol Biosynthesis” (2.34E-03). In the medium-exposure groups CDM and MDM, many of the same mechanisms as in the high-exposure groups were induced in the cod larvae, as suggested by the common molecules, with “Cytochrome P450 Panel - Substrate is a Xenobiotic (Human)” (3.07E-05) topping the IPA-Tox list, followed by “Aryl Hydrocarbon Receptor Signaling” (4.54E-05). This result clearly shows that components in the dispersed oil have triggered mechanisms known to be induced in animals after exposure to hydrocarbon contaminants.

## Discussion

The current microarray analysis suggests that chemically dispersed oil has lower transcriptomic effect on larvae of Atlantic cod than mechanically dispersed oil, based on the number of significantly affected transcripts and fold changes of a few transcripts. In this work we link the magnitude of transcriptional response to toxicity, especially for well-established biomarkers of mode of action of hydrocarbons such as the cytochrome P450 genes, even though we have not examined higher-level toxicity endpoints. Increasing knowledge, for example publications included in the Comparative Toxicogenomic Database (http://ctdbase.org), suggests this to be a valid assumption for transcriptional responses. Earlier studies suggest similar toxicity of chemically and mechanically dispersed oil in invertebrates and fishes [5,15,16], or more toxic effects of mechanically dispersed oil than of chemically dispersed oil on copepods and fish [17]. Clark et al. [5] showed for several organisms that the dispersants themselves did not alter the toxicity of oils, demonstrated by similar LC50 values for both chemically and mechanically dispersed crude oil. A similar finding was reported by Ramachandran et al. [18], who showed that the dispersant Corexit 9500 did not induce *cyp1a* in juvenile rainbow trout (*Oncorhynchus mykiss*). EPA has evaluated the contribution of dispersants on oil toxicity on shrimps and fish, including Corexit 9500A, which was used in the Gulf of Mexico 2010 incident, but were not able to see a universal trend [3]. By reducing the size of the oil droplets and increasing the aromatic hydrocarbon concentration, one would suspect that the dispersed fraction is more bioavailable to fish for accumulation via the gills and oral uptake [19]. However, conflicting evidence exists as to whether dispersed oil is more toxic than crude oil or untreated water-accommodated fraction of oil to fish. For example, Van Scoy et al. [20] showed that dispersant application significantly decreased hydrocarbon potency in Chinook salmon (*Oncorhynchus tshawytscha*) pre-smolts, whereas many studies suggest that the oil droplet fractions of oil dispersions increase the bioavailability and thereby the mechanism of toxicity of compounds of crude oil in fishes [18,21-23] or have only moderate effects on fish [14,24]. With a fold change cut-off of 1.5 and *p*<0.05, mechanically dispersed oil produced a much longer list of significantly affected transcripts than chemically dispersed oil. By comparing the significantly affected transcripts in larvae from the CDH and MDH exposure groups with the control in a PCA plot it also appears that mechanically dispersed oil is more toxic than chemically dispersed oil. One possible explanation for this finding is that the dispersant might have changed the characteristics of the oil droplets in a way that i) the dissolution rates of oil components into the water phase is lowered or ii) that the "stickiness" of oil droplet on fish larvae or rotifier (used as food for the larvae) surfaces is reduced. Since we obtained relatively comparable treatments in terms of oil concentrations, and the transcriptional effects are more pronounced for the mechanically dispersed oil than for the chemically dispersed oil, it is possible that the properties of the chemical dispersant decreases the exposure of cod larvae to oil components either through reduced dissolution of oil components, by reducing oil droplet fouling of cod larvae and/or reducing the uptake of oil droplets through food.

According to the microarray data, transcripts encoding cytochrome P450 system proteins were most strongly affected by the oil dispersions. *Cyp1a1* (or *cyp1a3*, equally matched), the transcripts showing the highest induction, was most severely affected in larvae in the MDH treatment group. This result is in line with numerous previous studies showing that CYP1A is easily induced in fish via the aryl hydrocarbon receptor (AHR) by components in the oil [25,26]. The induction of fish liver CYP1A has often been used as a molecular biomarker for exposure to petroleum hydrocarbons [26]. Several components of the crude oil can induce CYP1A, which is largely responsible for metabolism of PAHs and a variety of other toxic compounds [27-29]. Significantly elevated levels of *cyp1a* following exposure to the two oil dispersions were also determined by the RT-qPCR analyses. However, the more specific RT-qPCR analyses did not confirm that mechanically dispersed oil was more toxic based on the transcriptional levels of *cyp1a*, neither in the low, medium or high-concentration exposure larval groups. Instead they suggested that *cyp1a* was about 60-fold up-regulated by both types of oil dispersions. In a recent study in which cod larvae were exposed to dispersed oil or to the water-soluble fraction of oil (WSF), we observed a stronger induction of *cyp1a* in terms of fold change [14]. The relative levels of induction were greater following exposure to the dispersed oil, with a 300-fold up-regulation in the high-exposure group, compared to a 237-fold up-regulation in the high-exposure WSF group as suggested with the RT-qPCR data [14]. The reason for the lower induction levels of *cyp1a* transcription observed in the current study is unknown.

Interestingly, the three CYP1 transcripts quantified with RT-qPCR in the current study showed a different level of induction, with *cyp1a1*, *cyp1b1* and *cyp1c1* being 65, 12 and 8-fold up-regulated in larvae from the CDH group and 61, 10, and 8-fold up-regulated in larvae from the MDH group. Based on the microarray sequences used to design our PCR primers, the *cyp1a1* assay matched equally well against *cyp1a3* with BlastX searches, while the *cyp1c1* assay matched almost equally against *cyp1c2*, suggesting that more research are needed into the transcription of the different CYP1 genes and organ-specific function of their encoded proteins in cod.

In addition to the CYP1 genes, the aryl hydrocarbon receptor repressor (*ahrr*) transcript was also up-regulated in cod larvae for the high-exposure groups. The protein encoded by the *ahrr* transcript participates in the AHR signaling cascade, and is involved in regulation of cell growth and differentiation (GeneCards). AHRR represses the transcription of CYP1A1 by binding to the xenobiotic response element (XRE) sequence present in the promoter regulatory region of variety of genes. AHRR acts by recruiting ankyrin repeat, family A (RFXANK-like), 2 (ANKRA2), HDAC4 and/or HDAC5 to repress CYP1A1 in mammals (GeneCards). Several transcripts annotated to ankyrin genes were also up-regulated in cod larvae from the high-exposure groups, among them ankyrin repeat and btb domain containing 1 (*abtb1*). Histone deacetylase 1 (*hdac1*) was significantly down-regulated in larvae from both the CDH and MDH groups, while histone deacetylase 5 (*hdac5*) was significantly up-regulated in larvae from the MDH exposure group. These results suggest that both *cyp1a1* and *ahrr* mRNA inducibility is part of a mechanistic basis for resistance of fish larvae against compounds in dispersed oil, explaining the simultaneous induction of *cyp1a1* and *ahrr* mRNA. A similar finding has been reported for Atlantic tomcod (*Microgadus tomcod*), with a positive correlation between *ahrr* and *cyp1a1* mRNA levels in fish exposed to AH-responsive compounds [30]. Another explanation for this finding could also be that the dispersed oil mediated different effects in different organs, e.g. strong induction of *cyp1a1* transcription via AHR activation by aromatic hydrocarbons in liver, and effects via other mechanisms on *ahrr* transcription in other tissues. Organ-specific mechanisms cannot be studied in pooled whole larvae, representing a methodological limitation of using RNA from whole fish larvae for microarray examinations.

Mechanistic effects of contaminants can be studied with a number of tools. In this study we chose to use gene set enrichment analysis (GSEA) and pathway analysis with the Ingenuity Pathways Analysis (IPA) system. The GSEA data suggest that the two oil dispersions partly affected different cellular mechanisms, with several gene sets suggesting an effect on the proteasome complex. As part of the ubiquitin protein degradation system, the ubiquitin-protein ligases target specific proteins for ubiquitin-mediated proteolysis, and some of these genes potentially have a role in regulation of cell proliferation or differentiation (GeneCards). Components in the oil dispersions may therefore affect protein folding, and thereby activating ubiquitin-mediated proteolysis of misfolded proteins [31]. Comparing the two high-exposure groups CDH and MDH, in addition to the mentioned effect on the proteasome complex, the main difference between them seems to be that chemically dispersed oil specifically affected nucleosome assembly and DNA methylation by up-regulation of transcripts involved in these mechanisms, while mechanically dispersed oil mediated a down-regulation of the same gene sets. The mechanistic basis for this response is unclear, but this finding suggests that compounds in oil dispersions may affect epigenetic mechanisms in the developing cod larvae. Chromatin remodeling and altered DNA methyltransferase activity are key components of epigenetic regulation of gene expression, and these effects of dispersed oil should be studied more closely in follow-up investigations.

According to the IPA-Tox data, it appears that the oil dispersions have affected many well-known toxicological mechanisms, including aryl hydrocarbon receptor signaling, acute phase response proteins, oxidative stress, cell proliferation and nuclear receptor mediated responses. All of these represent well-known effects of toxic compounds in crude oil such as PAHs. Using a broader approach as shown in Additional files 4 and 5, the IPA Core Analyses suggest that chemically and mechanically dispersed oil share many of the top networks. Even when looking at transcripts that were uniquely affected in larvae from the different exposure groups, the data suggest a relatively similar mode of action in both exposure groups. As shown in Figures 4 and 5, transcripts common for the CDH and MDH groups, suggest that the dispersed oil mainly affected genes involved in DNA replication, recombination, and repair.

## Conclusions

In conclusion, this work suggests, if a significant altered number of affected genes can be used as a proxy to determine the exposure intensity, that chemically dispersed oil has lower toxic effects on Atlantic cod larvae than mechanically dispersed oil. Cytochrome P450 gene transcripts were most strongly affected in the exposed fish larvae. The main difference in mechanistic response between the two different oil dispersion treatments, was that chemically dispersed oil appears to have a stronger effect on nucleosome assembly and chromatin remodeling than mechanically dispersed oil, whereas the latter have a more pronounced effect on proteasome mediated protein proteolysis. Functionally, chemically and mechanically dispersed oil mainly affected similar mechanisms in the cod larvae, suggesting that the dispersant did not contribute strongly to the observed responses.

## Methods

### Materials and experimental set up

Atlantic cod (*Gadus morhua*) larvae were supplied from a commercial hatchery and hatched in the laboratory. Towards the end of the yolk sac stage on days 10–14 post hatch the larvae were exposed to dispersions of chemically and mechanically dispersed oil with similar oil concentrations and oil droplet sizes. The exposure period coincides with the first feeding period with the final absorption of the yolk sac and transition to external feeding.

Dispersions were generated using the method described by Nordtug et al. [32]. Three concentrations were used for both chemically and mechanically dispersed oil. The nominal amount of oil added was 0.25, 0.79 and 2.5 mg oil/L seawater. Analyses of PAHs were used to verify the actual exposure. For the chemically dispersed oil, the dispersant Dasic NS was premixed into the oil (4% w/w dispersant) before the oil was dispersed. In order to achieve similar oil droplet size distributions the energy input for generating the dispersion with Dasic NS was lower than for the purely mechanically generated dispersion [32]. The crude oil was artificially weathered by heating, creating a +200°C residue [33] before dispersed into filtered seawater (2 μm cartridge filter) through a series of nozzles yielding a constant flow of dispersion with a homogenous droplet size. Droplet size distributions were verified by Coulter Counter (Multisizer 3, with 100 μm aperture) and the average droplet size based on volume recorded in the outlet water was in the range 10–12 μm typically with at least 90% of the recorded oil mass was in the range 5 – 25 μm. For each dilution step a primary dispersion of 5 mg/oil per liter (nominal value) was continuously diluted with seawater using 3-way solenoid valves. Nordtug et al. [32] describe the procedures employed for generation of oil dispersions and dilutions, and the layout of the exposure chamber system. The exposure containers consisted of 5 L borosilicate glass bottles with bottoms removed and placed upside down in a water bath. Exposure solution (oil dispersion) and clean seawater (control) was added to the lower part of the exposure container through Teflon tubing (bore 1 mm). Water was drained from the surface through a 300 μm plankton mesh. The temperature (10°C ± 1°C) was controlled by submerging all exposure chambers into a water bath. The flow through in all exposure units was kept constant at 17,3 mL ± SD = 1.3 mL/min, corresponding to mean residence time of the water of approximately 4.5 hours. Dispersions were added by passive flow through thin inlet Teflon tubes (id =1 mm) and the flow was adjusted by changing the height of the inlet water column. A total number of 300 cod larvae were carefully introduced into each exposure chamber. The larvae were fed live rotifiers which were added in batches (5000 rotifiers per liter) three times a day together with algae (*Isochrysis galbana*, average concentration 0.5 – 1 mg wet weight/L). Four parallel exposure chambers were used in order to achieve biological replicates for every exposure concentration. The exposure design used in the experiment is given in Figure 1A. The cod larvae were exposed for 96 hours, counted and sampled.

### Larvae sampling and RNA extraction

At the end of the exposure period (on day 14), the cod larvae were immediately rinsed with distilled water and blotting paper and flash-frozen in liquid nitrogen, and stored at −80°C before RNA isolation. To ensure enough RNA was available for downstream transcriptomic analysis, 15 larvae were pooled together from each exposure unit. With this design, we had four biological replicates (n = 4) for each treatment, consisting of a total of 60 larvae. Due to an extensive sampling program and high lethality the available live larvae from one of the exposure units of the MDH and MDM groups was too few to be analyzed, leaving only 3 parallels for these groups. In total, RNA for transcriptomic analyses was isolated from 26 samples.

The larvae were thoroughly homogenized before RNA extraction using zirconium beads (4 mm) in a Precellys 24 homogenizer by ceramic beads CK28 (Bertin Technologies, Montigny-le-Bretonneux, France). Total RNA from Atlantic cod larvae was extracted using phenol chloroform extraction and Qiazol (Qiagen Hilden, Germany) with a modified isopropanol precipitation step previously described elsewhere [34]. The samples were subsequently treated with DNA-*free* (Ambion), according to the manufacturer's instructions and eluted in 50 μL RNase-free MilliQ H_2_O. The RNA was then stored at −80°C before further processing. RNA quality and integrity were assessed with the NanoDrop ND-1000 UV–vis Spectrophotometer (NanoDrop Technologies, Wilmington, DE, USA) and the Agilent 2100 Bioanalyzer (Agilent Technologies, Palo Alto, CA, USA). The RNA integrity number (RIN) was 7.8 ± 0.1 (mean ± SEM) evaluated for 15 samples, analyzed with the RNA 6000 Nano LabChip kit (Agilent Technologies, Palo Alto, CA, USA). RNA amplification was conducted using the TransPlex Whole Transcriptome Amplification WTA2 kit (Sigma Aldrich, St. Louis, MI, USA). The 260/280- and 260/230 nm ratios of the amplified RNA (cDNA) were 1.86 ± 0.00 and 2.14 ± 0.01 (mean ± SEM), respectively (n=26).

### Chemical analyses

Samples for semi-volatile organic compound analysis (approximately 800 mL each) were collected on baked glass bottles (1 L) and acidified with diluted hydrochloric acid (pH<2.0). A modified version of the US EPA guideline (US EPA, Method 3510C (1996). Separatory Funnel Liquid-Liquid Extraction. http://www.epa.gov/wastes/hazard/testmethods/sw846/pdfs/3510c.pdf) was used for extraction of water samples. Quantification of approximately 60 SVOCs in the C_10_ – C_22_ range included naphthalenes, PAHs, decalines and phenols was performed by use of Gas Chromatography/Mass Spectrometry (GC/MS) operated in selected ion monitoring mode. This method was also modified from a US EPA guideline (US EPA, Method 8270D (2007) (Semivolatile Organic Compounds by GC/MS. http://www.epa.gov/wastes/hazard/testmethods/sw846/pdfs/8270d.pdf).

### Microarray analyses

The microarray gene expression screening study was conducted using a 12 plex 135K Nimblegen custom-made gene expression array (Atlantic Cod Oligonucleotide 135K Array V1). This microarray was designed using cod expressed sequence tags (ESTs) available from the GAFFA database (http://genofisk.cbu.uib.no). A total of 42 111 cod sequences from the GmE100215 Atlantic cod EST assembly representing 26 065 contigs (assembled ESTs representing the same mRNA transcript) and 18 067 singletons (single ESTs) were selected for microarray probe design. Of the selected contigs, 25 749 had Basic Alignment Search Tool X (BlastX) hit E-values <1 against known protein sequences in the RefSeq database (http://www.ncbi.nlm.nih.gov/RefSeq/) [35], and 316 were predicted to contain conserved protein domains using predicted protein Blast against the Pfam database (http://pfam.sanger.ac.uk/) [36]. In addition, singletons with a minimum bit score of 45 to a UniRef90 cluster [37] (http://www.uniprot.org) were included. Three different 60-mer DNA oligo probes was designed for each transcript. The probes were designed and printed by Nimblegen using the Nimblegen probe design pipeline previously published (Roche Nimblegen Probe design Fundaments 2008). Of the 44 132 sequences used as input in the probe design pipeline, 2 021 transcripts were discarded either due to presence of overlapping probes and possible cross hybridization, or because no satisfactory probe design was possible. In total, 125 826 probes were printed on each array. Array hybridization of amplified cDNA samples was conducted by Roche Nimblegen (Reykjavik, Iceland). The hybridization, data extraction and quantile normalization protocol has previously been described in detail elsewhere (Nimblegen Arrays User’s Guide: Gene Expression Arrays v5.0, 2010 Roche Nimblegen, Inc.). Gene calls of triplicate probe expression values were generated using the Robust Multichip Average (RMA) algorithm as described by Irizarry et al. [38,39]. Probe calls (collapsed probes) with large variation (SE > 0.8) between the triplicate probes were removed from the dataset prior to downstream analysis using the J-Express Pro microarray analysis software (http://jexpress.bioinfo.no). BlastX sequence predictions, gene ontology terms and gene symbols were retrieved using the Blast2GO control suite (http://www.blast2go.com). Sequence identity E-value cut off <E-6 was used for KEGG and GO annotation yielding 36 946 probes with sequence description, 27 563 sequences assigned to GOs and 6 784 sequences given KEGG enzyme identity numbers (EC numbers). The microarray data have been submitted to the ArrayExpress EBI database according to the MIAME guidelines (ArrayExpress accession number E-MTAB-1372).

### Quantitative real-time RT-qPCR

In total 20 genes were quantified with RT-qPCR. PCR primer sequences used for the quantification of the transcriptional levels of the target genes as well as the reference genes β-actin (ACTB), elongation factor 1 alpha (EF1A), ubiquitin (UBI) and ribosomal protein R4 (RPL4), are shown in Table 3. BlastX or BlastN was used to determine PCR assay specificity. The reaction specificity of each assay was verified by observing a single peak in the melting curve. Nine of these genes were selected in order to verify the microarray data. Seven other target genes were selected as generic stress markers.

**Table 3:** **GenBank accession numbers, PCR primers, amplicon sizes and PCR efficiencies of the RT**-**qPCR assays used in the current experiment**

**Gene**	**Accession no.**	**Forward primer**	**Reverse primer**	**Amplicon size(bp)**	**PCR efficiency**
CYP1A	EX725014	CCTTGACCTCTCGGAGAAAGAC	CGCCCCGCTAGCTATAGACA	146	1.85
CYP1B1	GmE090818c16636	CAGATGCCCCACATCACCTT	GATCGCCTTGTCTCCGTTCA	108	2.10
CYP1C1	GmE100215i31189	GTCAACCAGTCGTCCGTGAA	CGTTGTTCGTGAGGTCCTTGT	115	2.02
AhR2	EX728781	CAACCGGCGGTCCACAT	GCACCATGCAGTTGCCAGTA	132	2.08
AHRR	F2Z8C0H01CDEIU	GAGGTGTTTGTCTCCCGTCTCT	TTCACCAGGTCCACGCAGTA	77	2.06
GST pi	EX730032	GTCCCCCTGCTGCCATTC	CCTCCATACACCGCCACCTA	126	1.85
p53	EX723548	CGCTGCTGCTGAACTTCATG	GGATGGCTCTCCGGTTCAT	63	1.86
RAD51		AACCTAGCATCCCGCTCAAA	TGGTCAGGACAACTGGGATGT	91	1.89
IGF1		CTCTCCAAATCCGTCTCCTGTT	AGATGCGGGCAATGTCACAT	101	1.99
IGFBP1		GTGAGTCCTCCCTGGATGGA	CTCGACCCCAGGATCTTCTTC	79	2.02
Ferritin	EX726260	GCGTCTAGCTGGACCCCTTA	GGGCGACTACATCAGCAACC	101	2.08
Transferrin	EX722617	ACGACGGGTCAACTCGTTTG	AGTCACTCGGGCACAATGTG	139	1.83
HSP70	EX741726	CATGACATCGTCCTGGTTGGT	CGTAGGCCACAGCTTCATCA	121	1.84
HPX (WAP65-2)	GmE100215i27905	CGCCTGCTCTACATCGTCAA	CGTAGCGTCCAGTGGTCCTT	129	1.89
MCM2	GmE100215i14387	TGGAGGACGACGTGAACATG	TCGTTGTTGTCGCGTTTGAA	130	1.77
CDCA7	GmE100215i27789	GCATCTGCACCAACGTGAAG	CCCCCACACACTCTGGGTTA	120	2.02
EF1A	EX722124	CGGTATCCTCAAGCCCAACA	GTCAGAGACTCGTGGTGCATCT	93	1.97
B-actin	EX739174	CACAGCCGAGCGTGAGATT	ACGAGCTAGAAGCGGTTTGC	95	1.93
Ubiquitin	EX735613	GGCCGCAAAGATGCAGAT	CTGGGCTCGACCTCAAGAGT	69	1.93
RPL4	EX725958	GGTGCCATACAGCTGATCCA	CCAGGCATCACACTGCAGAA	123	1.83

RT-qPCR was conducted as previously described by Olsvik et al. [40]. Briefly, a two-step real-time RT-PCR protocol was used to quantify the transcriptional levels of the 20 target genes in the larvae. The RT reactions were run in duplicate on a 96-well reaction plate with the GeneAmp PCR 9700 machine (Applied Biosystems, Foster City, CA, USA) using TaqMan Reverse Transcription Reagent containing Multiscribe Reverse Transcriptase (50 U/μL) (Applied Biosystems, Foster City, CA, USA). Two-fold serial dilutions of total RNA were made for efficiency calculations. Six serial dilutions (1000–31 ng) in triplicates were analyzed in separate sample wells. Total RNA input was 500 ng in each reaction for all genes. No template controls (ntc) and RT-controls were run for quality assessment. RT-controls were not performed for every individual sample, but were run for each assay or gene. Reverse transcription was performed at 48°C for 60 min by using oligo dT primers (2.5 μM) for all genes in 50 μL total volume. The final concentration of the other chemicals in each RT reaction was: MgCl_2_ (5.5 mM), dNTP (500 mM of each), 10X TaqMan RT buffer (1X), RNase inhibitor (0.4 U/μL) and Multiscribe reverse transcriptase (1.67 U/μL) (Applied Biosystems).

Twofold diluted cDNA (1.0 μL cDNA from each RT reaction) was transferred to 384-well reaction plates and the qPCR run in 10 μL reactions on the LightCycler 480 Real-Time PCR System (Roche Applied Sciences, Basel, Switzerland). Real-time PCR was performed by using SYBR Green Master Mix (LightCycler 480 SYBR Green master mix kit, Roche Applied Sciences), which contains FastStart DNA polymerase, and gene-specific primers (500 nM of each). PCR was achieved with a 5 min activation and denaturizing step at 95°C, followed by 45 cycles of a 15 s denaturing step at 95°C, a 60 s annealing step and a 30 s synthesis step at 72°C. Target gene mean normalized expression (MNE) was determined using a normalization factor calculated by the *geNorm* software based on the three selected reference genes (ACTB, EF1A and UBI) [41].

### Statistics

J Express software (http://jexpress.bioinfo.no) was used to analyze the microarray data, including to generate gene lists and for functional analysis using Gene Set Enrichment Analysis (GSEA). The functional pathway analyses were generated through the use of IPA (Ingenuity Systems, http://www.ingenuity.com). The GraphPad Prism 5.0 software (GraphPad Software, Inc., San Diego, CA, USA) was used for statistical analyses of the RT-qPCR data. ANOVA was used to search for treatment effects at the transcriptional level. Dunnett’s multiple comparison and Newman Keuls posthoc tests were used to compare the exposed groups against the control or for comparison between exposure groups. A significance level of P < 0.05 was used for all tests.

## Competing interests

The authors declare no competing interests.

## Authors' contributions

TN designed the exposure system and initiated the study. TN and BHH conducted the Atlantic cod larvae exposure experiment, and did the chemical analyses. KKL was responsible for design and analyses of the microarray study. PAO was in charge of the transcriptomics work and drafted the manuscript. All authors have read and approved the final manuscript.

## Supplementary Material

Additional file 1 **Concentrations (in μg/L) of ∑ PAHs in the exposure media during the experiment.** Values are given as average ± standard deviation (N=8). Click here for file

Additional file 2 **ANOVA generated gene lists.** Gene lists generated with ANOVA analysis from the six groups of larvae (CDH, CDM, CDL, MDH, MDM and MDL), with sequence IDs, sequence descriptions, gene names used for functional analysis, P-values and fold changes. ANOVA. P<0.05. Click here for file

Additional file 3 **Top ranked gene sets.** Top ranked gene sets in larvae from all exposure groups compared to the control group, as analyzed with Gene Set Enrichment Analysis (GSEA) and Ingenuity Pathway Analysis (IPA). Annotations as of June 2011. Click here for file

Additional file 4 **Top affected IPA networks.** Top affected IPA Core Analyses networks in the cod larvae exposed to the highest concentration of chemically dispersed oil (CDH group) and mechanically dispersed oil (MDH). All molecules in each group included. Click here for file

Additional file 5 **IPA Compare networks.** In an attempt to identify unique and common molecules across the gene lists the IPA-Compare function was applied. Additional file 5 shows the associated functions of the top networks as suggested by IPA Core Analysis in significantly affected transcripts in cod larvae exposed to the different exposure treatments. Only molecules uniquely affected in each group included. Click here for file
